# Lubrication Performances of Polyalkylene Glycols at Steel Interface under External Electric Fields

**DOI:** 10.3390/nano12122067

**Published:** 2022-06-15

**Authors:** Xiangyu Ge, Xiaodong Wu, Qiuyu Shi, Wenzhong Wang

**Affiliations:** 1 School of Mechanical Engineering, Beijing Institute of Technology, Beijing 100081, China; wuxd_me@163.com (X.W.); wangwzhong@bit.edu.cn (W.W.); 2 State Grid Smart Grid Research Institute Co., Ltd., Beijing 102209, China

**Keywords:** lubrication, friction, external electric field, insulating oil

## Abstract

This work studied the lubrication performances of polyalkylene glycols, which are insulating oils, at the steel interface under external electric fields. The results show that external electric fields greatly affect the lubrication performances of polyalkylene glycols, and there is an optimal voltage (−1.0 V) for the improvement in friction reduction performance. The surface analysis and experiment results indicate that the polyalkylene glycol adsorption film and the reduction in the amount of Fe_x_O_y_ and FeOOH in the tribochemical film contribute to improved friction performance under the negative voltage condition. This work proves that the lubrication performances of insulating oils can be affected by external electric fields as well. A lubrication model was proposed, hoping to provide a basic understanding of the lubrication mechanisms of ether-bond-containing insulating oils in the electric environment.

## 1. Introduction

With the dependence on technology, more and more mechanical parts operate under external electrical fields (EEFs), such as the head/disk interface in ultrahigh-density magnetic storage systems, the charged movable interface in micro/nanoelectromechanical systems, electrified trains, motors, wind turbine generators, aircraft propulsion systems, and slip rings [1,2,3,4]. The electrical conditions can lead to the variation in lubricating performances [5] and result in premature bearing and motor failure, such as in electric vehicles [6]. Therefore, it is of great significance to study the lubrication performance under an electric field.

Since Guruswamy and Bockris first coined the term “Triboelectrochemistry” in 1980 [7], lubrication under EEFs has been vigorously developed based on the in-depth study of lubrication mechanisms and the urgent needs of industry [8,9,10]. Yoshitsugu et al. [11] found that there is a threshold voltage for liquid crystal when used as a lubricant at the steel/steel interface under boundary lubrication. When the applied voltage is lower than the threshold value, the coefficient of friction (COF) is not affected, and, once the threshold voltage is exceeded, the COF decreases, apparently. Luo et al. [12,13] studied the influence of EEFs on film thickness in thin film lubrication. Oleic acid and other additive-containing oils were used as lubricants, and it was found that a larger EEF during sliding results in a thicker film and, hence, a larger COF value. In recent years, a large number of studies have been conducted to study the lubrication performances of ionic liquids (ILs) under EEFs due to their sensitivity to EEFs [14,15,16,17]. It was proved that, when employed as lubricants under EEFs, ILs can form a classic, self-assembled, double-layer structure at the sliding interface [18]. Friction reduction and antiwear performances are ultimately determined by the synergistic effect of the physical adsorption and tribochemical reaction of ILs. However, due to the presence of ions, corrosion and oxidation may restrain the further application of ILs [19].

It is noted that most of the studies focused on lubricants with high electrical conductivity or introducing strong electrolytes as additives. The studies on the specific lubrication performances of insulating oils and weak electrolytes under EEFs focused on the film-forming characteristic, while the friction property was rarely studied. Oil-based lubricants play a dominant role in industrial lubrication, and most lubricating oils are insulating oils, such as polyalkylene glycol (PAG), poly-α-olefin (PAO), and polyethylene glycol (PEG). Therefore, the friction performance and the corresponding mechanisms of insulating oils under EEFs need to be revealed. This work studied the friction performance of PAGs as lubricants at the steel interface under EEFs, and the mechanism of improved lubrication performance was revealed.

## 2. Materials and Methods

### 2.1. Materials and Experimental Methods

**Figure 1:**
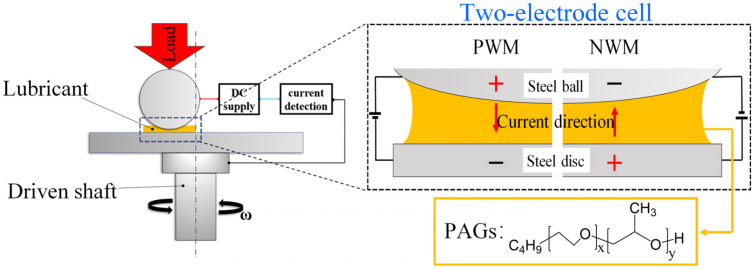
Schematic diagram of the experimental facility under EEFs.

The PAGs used in this work were provided by Dow Chemical Co., Ltd., Mitland, Michigan, US, including 50-HB-170 (PAG170), 50-HB-260 (PAG260), and 50-HB-660 (PAG660). All these PAGs had a purity of over 99% and were used without further treatment. The PAG viscosity was tested at room temperature by a rheometer (MCR101, Anton Paar, Graz, Austria). Friction and wear tests were performed on a universal micro-tester (UMT-3, Bruker, Karlsruhe, Germany). An additional device based on the two-electrode cell principle was designed to provide different EEFs for both ends of the friction pair operating on UMT-3 (Figure 1). The fixed ball (GCr15, Ø 10 mm, surface roughness (*Ra*) ≈ 10 nm) was loaded on a rotating disc (GCr15, *Ra* ≈ 25 nm). The COF was processed and presented automatically. Before the test, the balls and discs were ultrasonically cleaned with ethanol, acetone, and pure water (15 min for each reagent).

For each test, a quantitative liquid (30 μL) was dropped into the contact area of the friction pair. The loading force ranged from 2 to 4 N, corresponding to the maximum Hertz contact stress in the contact area of approximately 466 MPa (4 N). The sliding velocity ranged from 30 to 100 mm/s. The external power supply was directly connected to both ends of the friction pair to apply voltage ranging from −2.0 to +2.0 V. To study the effect of applying EEFs on the lubrication performance, two wiring modes were used in the experiment. As shown in Figure 1, the positive wiring method (PWM) represented the method of connecting the positive pole of the power supply to the ball and the negative pole to the disc, and the applied voltage was denoted as a positive value such as +1.0 V. The negative wiring method (NWM) represented the wiring method of connecting the negative pole to the ball and the positive pole to the disc, and the applied voltage was denoted as a negative value such as −1.0 V. Each test was repeated three times, and the measurement accuracy of COF was ±0.001. To obtain more accurate test results, the levelness of the loading platform and the verticality of the loading device were adjusted so that the same COF value could be obtained while the platform rotated clockwise and counterclockwise. All tests were conducted at room temperature with a relative humidity of 10–30%.

### 2.2. Surface Analysis

After the experiment, the diameter of the wear spot (WSD) and surface roughness of the ball’s wear spot were measured by a microscope and a white light interferometer (Nexview, Zygo Lamda, Middlefield, CT, USA), respectively. The surface morphology of the wear surface of the steel disc was observed by a scanning electron microscope (SEM, S4800, Hitachi, Tokyo, Japan). An X-ray photoelectron spectroscope (XPS, PHI QUANTERA-II SXM, Ulvac-Phi, Chigasaki, Japan) and a Raman spectrometer (LabRAM HR Evolution, HORIBA Scientific, Paris, France) were used to characterize the chemical state of the wear surfaces.

## 3. Results and Discussion

### 3.1. Lubrication Performances

**Figure 2:**
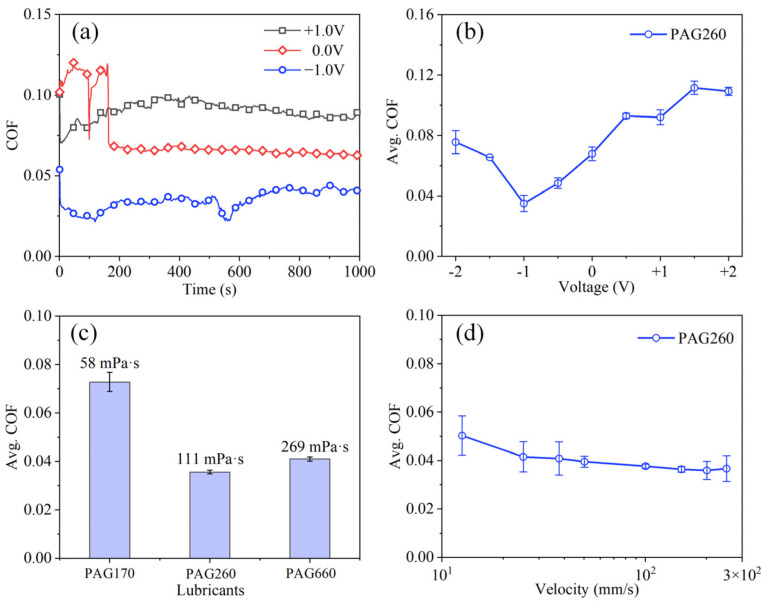
(**a**) COFs with the lubrication of PAG260 under ±1.0 V voltages, (**b**) COF curve of PAG260 varying with voltages, (**c**) comparison of lubricating oils with different viscosities under the optimal voltage (−1.0 V), (**d**) COF evolution with velocity under the optimal voltage (−1.0 V).

To study the lubrication performances of PAGs under EEFs, the COFs with the lubrication of PAG260 were measured under ±1.0 V voltages, and those with an EEF were also measured to make a comparison (Figure 2a). The results show that, except for the large fluctuation in the early stage with the EEFs, the COF curves under both +1.0 V and −1.0 V voltage reached a relatively stable state with small fluctuation in a short time. The average COF value in the stable lubrication state (600–1000 s) was taken for comparison. The results show that the COF value (~0.089) under the +1.0 V voltage was approximately 39% larger than that with no EEFs (~0.064). In contrast, the COF value (~0.04) under the −1.0 V voltage was approximately 37.5% smaller than that with no EEFs. These results prove that the lubrication performances of PAG260 were affected by EEFs, and the NWM condition decreased the COF while the PWM condition increased the COF compared with that with no EEF. To further study the influence of EEFs on the lubricity of PAGs, PAG260 was tested in a range of voltages from −2.0 to +2.0 V (Figure 2b). The results show that the COF value increased from 0.0679 to 0.1093 with the increase in voltage from 0 to +2.0 V when the PWM condition was employed. Regarding the NWM condition, the COF value first decreased from 0.0679 to 0.0361 with the reduction in voltage from 0 to −1.0 V, then increased from 0.0361 to 0.0756 with the further reduction in voltage from −1.0 to −2.0 V. This result shows that the optimal voltage for PAG260 to achieve the minimum COF value was approximately −1.0 V, and the minimum COF value was approximately 0.0361.

To explore the influence of lubricating oil viscosity on lubricity under optimal voltage, further friction lubrication experiments were conducted on PAGs with different viscosities (Figure 2c). The results show that, under the optimal voltage (−1.0 V), the friction performance of PAG260 was better than the other PAGs used in this work. Thereafter, the influence of sliding velocity on the lubrication performances of PAG260 under the optimal voltage (−1.0 V) was studied (Figure 2d). The results show that, when the velocity increased from 12.5 to 200 mm/s, the COF decreased from 0.05 to 0.035, while the COF increased from 0.035 to 0.036, and the velocity further increased from 200 mm/s to 250 mm/s. These results indicate that there is an optimal viscosity and sliding velocity for PAGs to exhibit improved lubrication performance.

**Figure 3:**
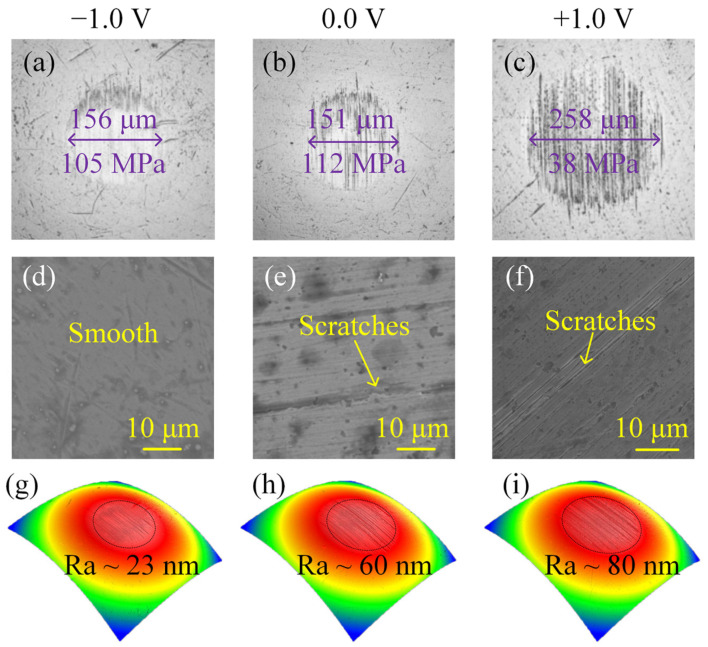
Images of wear surfaces after being tested under different EEFs, (**a**–**c**) wear spots on the balls, (**d**–**f**) surface topographies of the wear surfaces on the discs, (**g**–**i**) *Ra* of the wear surfaces on the balls.

To analyze the lubricating mechanisms under EEFs, WSDs, morphologies, and the surface roughness of the wear surfaces were observed (Figure 3). Regarding the WSD, the value (~258 μm) obtained under the +1.0 V voltage was approximately 71% larger than that (~151 μm) obtained in the absence of an EEF, indicating that the PWM condition aggravates the wear. In contrast, the −1.0 V condition only had a slight effect on the wear performance when there was a WSD value of approximately 156 μm. This result indicates that the WSDs were not the key factor that resulted in the friction reduction under −1.0 V voltage, because the contact pressures for the −1.0 V and 0.0 V conditions were nearly the same. The surface morphology results show that the ball and disc’s wear surfaces obtained under −1.0 V voltage were relatively smooth, and only shallow grooves could be observed, while, in the absence of EEFs and the +1.0 V voltage, the presence of clear scratches and deep grooves could be observed on the wear surfaces. According to the results of the 3D white-light interferogram, the surface roughness of the three cases was 23 nm (−1.0 V), 60 nm (0.0 V), and 80 nm (+1.0 V). These results indicate that EEFs affect the wear level of the materials. The NWM condition only slightly affected the wear performance, while the PWM condition resulted in severe wear when compared with the condition with no EEF.

### 3.2. Discussions

To determine the lubricating state of PAG260 under the −1.0 V condition, the H−D formula (Equation (1)) [20] was used to estimate the fluid film thickness in a stable lubrication state:

(1) $$h_{c}=2.69\frac{Q^{0.53}RS^{0.67}}{M^{0.067}}\left(1-0.61e^{-0.73c}\right)$$

where $Q=\kappa E^{\prime }$, $S=\eta u/E^{\prime }R$, $M=w/E^{\prime }R^{2}$, and *κ* is the viscosity-pressure coefficient of PAG260 (12.3 × 10^−9^ Pa^−1^) [21]. *η* is the dynamic viscosity of PAG260 (~111 mPa·s), *u* is the average relative sliding velocity of the friction pair (50.24 mm/s), *w* is the normal load (2 N), *c* is a constant coefficient (generally 1), and *E*′ is the equivalent elastic modulus of surface material, which was calculated by Equation (2):

(2) $$E^{\prime }=2/\left[\left(1-v_{1}^{2}\right)/E_{1}+\left(1-v_{2}^{2}\right)/E_{2}\right]$$

where *E*_1_ and *E*_2_ are the elastic modulus of GCr15 (208 GPa), and *v*_1_ and *v*_2_ are the Poisson’s ratio of GCr15 (0.3). To obtain the effective contact radius (*R*) of the wear spot on the ball, the diameter of the wear spot on the ball was assumed to be the Hertz elastic contact deformation under normal load; that is, the diameter of the wear spot on the ball (156 μm) was described by Equation (3):

(3) $$\frac{d}{2}={\left(\frac{3Rw}{4E^{\prime \prime }}\right)}^{\frac{1}{3}}$$

Therefore, the effective contact radius was estimated by Equation (4):

(4) $$R=\frac{E^{\prime \prime }d^{3}}{6w}$$

where *E*″ is the effective comprehensive elastic modulus of the friction pair material,

(5) $$E^{\prime \prime }=1/\left[\left(1-v_{1}^{2}\right)/E_{1}+\left(1-v_{2}^{2}\right)/E_{2}\right]$$

In summary, the fluid film thickness of PAG260 in the stable lubrication stage was *h*_c_ ≈ 113.9 nm. The corresponding lubrication state was divided by the film thickness ratio (Equation (6)):

(6) $$\lambda =\frac{h_{c}}{\sqrt{\sigma_{1}^{2}+\sigma_{2}^{2}}}$$

where *σ*_1_ (~23 nm) and *σ*_2_ (~47 nm) are the surface roughness of the wear zone of the ball and disc, respectively. Then, *λ* was calculated as approximately 2.18, indicating a mixed lubrication state in the stable lubrication stage, namely, the boundary film (tribochemical film) and the fluid film existed at the interface at the same time. This result explains the phenomenon where the COF varied with the PAG viscosities and sliding velocities (Figure 2c,d). The thickness of the fluid film varied with the PAG viscosities and sliding velocities, which influenced the COF value and resulted in the existence of an optimal PAG viscosity and sliding velocity for PAGs to exhibit improved lubrication performance.

**Figure 4:**
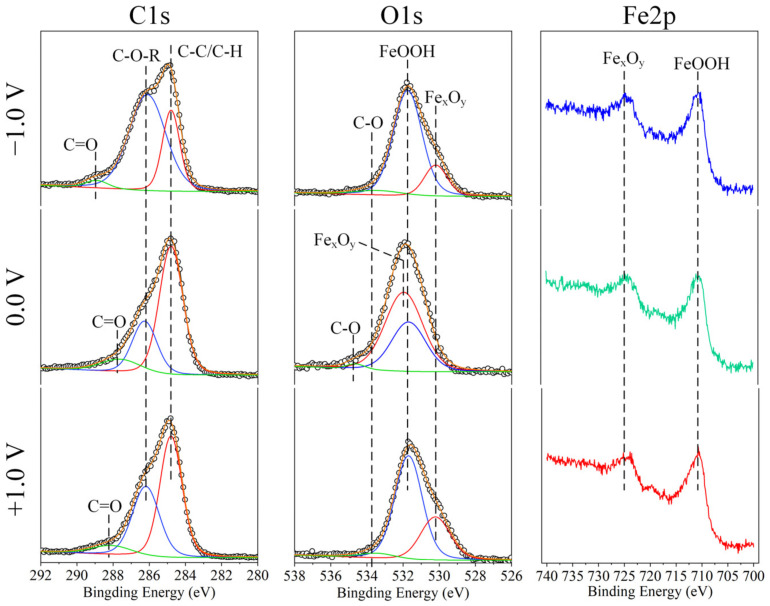
XPS results on disc scratch under different EFF conditions.

To explore the lubrication mechanism of PAGs under EEFs, the chemical composition of the wear surface on the disc was detected by XPS (Figure 4). In the C1s spectra, the bonds detected on the wear surface were similar for all the EEF conditions, including C−O/C−H (284.8 eV), C−O−R (286.2 eV), and C=O (288.1/287.6/288.8 eV) [22,23], which may derive from the adsorption of PAG on the surface. In the O1s spectra, the peaks ranging from 530 to 532 eV indicated the generation of iron oxides by tribochemical reactions, and the peaks at 533.4 and 534.6 eV indicated the generation of carbon−oxygen compounds. The peaks of Fe2p at both 711.5 and 724 eV further indicated the generation of FeOOH and Fe_x_O_y_ under all the EEF conditions [24]. According to the XPS results, tribochemical reactions and adsorption effects occurred during the friction process, and the products on the wear surfaces were similar for various EEF conditions; therefore, the types of products are not the key factors that affect the lubrication performances.

Thereafter, the proportion of products was discussed. Table 1 provides the proportion of carbon- and oxygen-containing compounds according to C1s and O1s spectra, respectively. The results show that the proportion of ether bonds (C−O−R) on the disc significantly increased when NWM was applied, indicating the increase in the degree of PAG adsorption to the disc. For the NWM condition, the disc was connected to the positive pole of the power supply, making the negatively charged ether bonds easily adsorb to the disc and form a PAG adsorption layer, and this may result in improved lubrication performance. Further, in the absence of EEFs, the proportion of Fe_x_O_y_ accounted for approximately 60% of the total content, while the main product was changed to FeOOH (~70%) for both the PWM and NWM conditions. This indicates that the EEF promoted the transformation of Fe_x_O_y_ to FeOOH through electrochemical reactions. However, the product proportion on the wear surface was similar for both the PWM and NWM conditions. Therefore, it is not the key factor that affects the lubrication performances under EEF conditions.

**Figure 5:**
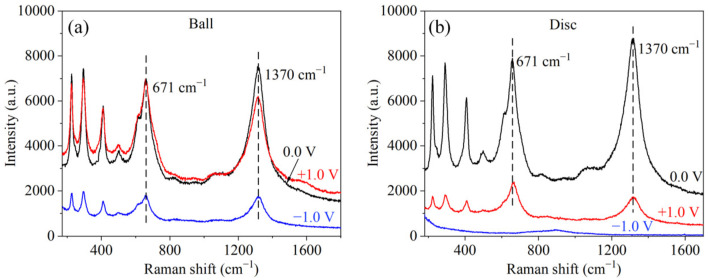
Raman spectra of the wear surfaces, (**a**) the ball and (**b**) the disc.

To further explore the mechanism, Raman analysis was used to study the number of products on the wear surfaces lubricated with PAG260 under +1.0, 0.0 and −1.0 V conditions. The main peaks detected at 671 and 1370 cm^−1^ were assigned to FeOOH and Fe_x_O_y_, respectively (Figure 5) [25]. With no EEFs applied, both the FeOOH and Fe_x_O_y_ were detected on the ball, and the amount of Fe_x_O_y_ was slightly larger than that of FeOOH. In contrast, the amount of FeOOH was slightly larger than that of Fe_x_O_y_ in the case of the PWM condition. A similar phenomenon could be observed on the discs for both the PWM and no EEF conditions. Notably, the product amount on the disc was much less than that on the ball for the PWM condition. In the case of the NWM condition, only a small amount of FeOOH and Fe_x_O_y_ was detected on the ball, while the amount of FeOOH and Fe_x_O_y_ was undetectable on the disc. This may be because the ball had constant contact in the experiment, but the disc only had intermittent contact. The tribochemical period was much shorter for the disc than for the ball, resulting in fewer (PWM condition) and no obvious (NWM condition) tribochemical compounds being formed and detected on the discs. This result indicates that the reduction in the amount of FeOOH and Fe_x_O_y_ generated on the ball and disc under the NWM condition may improve the lubrication performance.

**Figure 6:**
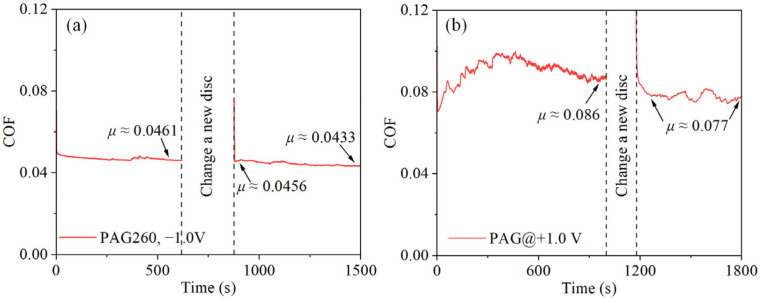
Disc replacement experiment, (**a**) −1.0 V and (**b**) +1.0 V.

To verify this deduction, experiments were designed and conducted. Firstly, the friction experiment was conducted under the NWM condition (−1.0 V), as before. When the lubrication state was stable, the experiment was suspended, and the disc was changed to a fresh one. Then, the experiment was restarted on the same wear spot on the ball. The result showed that the COF slightly decreased from 0.0461 to 0.0456 after disc replacement and was maintained at 0.0433 at the end of the test (Figure 6a). This result indicates that the friction performance can be improved by removing the product from the disc. However, because of the undetectable number of products on the disc under the NWM condition, the improvement in lubrication performance was limited. According to XPS results, the product amount on the disc under the PWM condition was more than that under the NWM condition. Therefore, this experiment was conducted under the PWM condition, and the result showed that the COF apparently decreased from 0.086 to 0.077 after disc replacement (Figure 6b). This result supports our deduction that the fewer electrochemical reaction products on the disc, the more favorable it is for lubrication performances.

**Figure 7:**
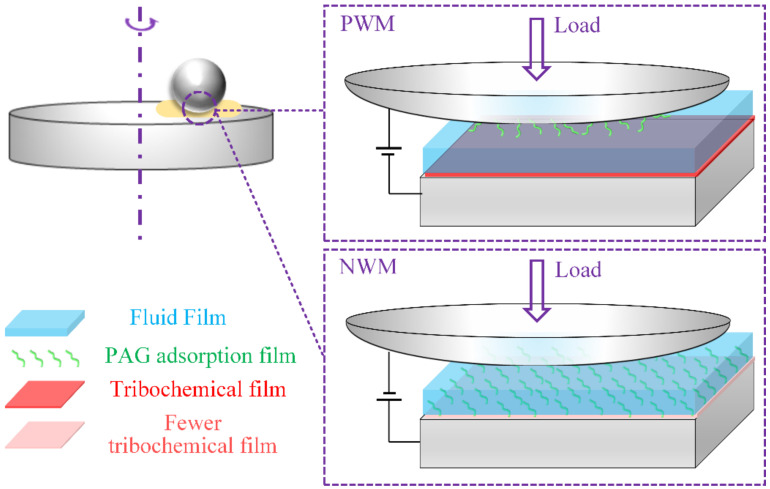
Diagram of the lubricating mechanism for PAGs under EEF conditions.

According to the above analysis, a lubrication model for PAGs under the EEF condition was proposed (Figure 7). The film-forming character of the PAG adsorption film and tribochemical film was different for various EEFs. In the case of the NWM condition, the steel disc was connected to the positive pole of the power supply, thereby the PAG molecules could be adsorbed to the disc through the negatively charged ether bonds and form an adsorption film. During the friction process, one point of the wear track on the disc was in an intermittent contact state. When the ball and the point of the disc were in contact, the PAG adsorption film protected the surfaces and contributed to low friction; when the point was not in contact, the worn PAG adsorption layer could recover under the EEF condition and wait for the next round of contact. Therefore, continuous and effective protection could be performed on the surfaces, resulting in improved lubrication performances and fewer tribochemical reactions occurring on the surfaces. Furthermore, the reduction in the amount of Fe_x_O_y_ and FeOOH in the tribochemical film was conducive to lower friction, which was proved by the experiment results. This is a new finding because FeOOH is considered to facilitate friction reduction in general [26,27]. However, under the PWM condition, the positive pole of the power supply was connected to the ball. Thus, the PAG molecules were adsorbed to the steel ball driven by EFF. Since the ball was in a constant contact state during the friction process, the PAG molecules remained exposed to severe wear, and it was difficult to form a stable PAG adsorption film. Therefore, the lubrication performance was not improved.

**Figure 8:**
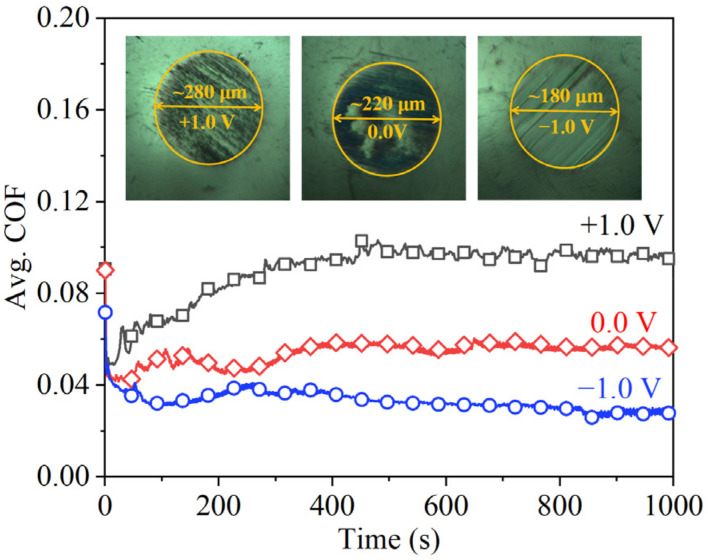
COFs and WSDs with the lubrication of PEG400 under various EEFs.

This study shows that ether bonds may facilitate improvement in lubrication performances. Therefore, PEG, which has ether bonds as well, was tested under EEF conditions. The results show that PEG exhibited similar results to PAGs (Figure 8), which is that the NWM condition facilitated the improvement in lubrication performances. Therefore, the lubrication mechanism proposed for PAGs might be suitable for other ether-bond-containing lubricants. Despite other factors that must be matched for industrial application, this result demonstrates the potential of such oil-based lubricants for lubrication in an electrical environment.

## 4. Conclusions

This work studied the lubrication performances of PAGs at the steel interface under EEFs. It was concluded that (1) the lubrication performances of ether-bond-containing insulating oils can be affected by EEFs. (2) The friction increases with increasing voltage under the PWM condition and decreases first and then increases with the voltage variation under the NWM condition. (3) There is an optimal voltage (−1.0 V) and PAG viscosity (~111 mPa·s) for the improvement in friction reduction performance under NWM conditions. (4) The friction decreased with the sliding velocity increasing from 12.5 to 250 mm/s. (5) The improved lubrication performances under the NWM condition were attributed to the PAG adsorption film and the reduction in the amount of Fe_x_O_y_ and FeOOH in the tribochemical film. Moreover, a lubrication model was proposed, hoping to provide a basic understanding of the lubrication mechanisms of ether-bond-containing liquids in the electric environment.

## Figures and Tables

**Table 1:** Product proportion according to C1s and O1s spectra.

Voltage (V)	Formula (C)	Proportion (%)	Formula (O)	Proportion (%)
+1.0 V	C−O/C−H	54.22	FeOOH	66.07
	C−O−R	38.12	Fe_x_O_y_	29.66
	C=O	7.66	C−O	4.27
0.0 V	C−O/C−H	62.29	FeOOH	35.67
	C−O−R	26.8	Fe_x_O_y_	62.34
	C=O	10.91	C−O/C=O	1.99
−1.0 V	C−O/C−H	30.71	FeOOH	76.41
	C−O−R	65.60	Fe_x_O_y_	18.01
	C=O	3.69	C−O	5.58

## Data Availability

Not applicable.
